# Danger from Hypodermic Injections

**Published:** 1877-08

**Authors:** E. Fletcher Ingals


DANGER FROM HYPODERMIC INJECTIONS.—*By E. Fletcher Ingals, M.D.*—I have often used hypodermic injections of morphia, and always with good results, until a few weeks since, when I obtained alarming results from the administration by this method of one-fourth of a grain of morphia.

The patient, in consequence of continuous watching with sick children, had become debilitated, and as a result, suffered at times from severe pains of a neuralgic character.

I was called in the night to see her in one of these attacks. The pain had commenced about twelve hours previously, and with frequent exacerbations, had steadily increased in severity until it had become unbearable.

I dissolved one-fourth of a grain of morphia in pure water, and administered it under the integuments on the outer side of the arm. Within a few seconds the breathing became stertorous, the pulse failed, the lips and countenance became livid, and the eyes were set; respiration ceased, the radial and cardiac pulsations were lost, and the heart sounds could not be distinguished. The woman was to all appearances dead. How long this condition continued I cannot tell; it seemed an age, but was probably only ten or fifteen seconds, for by prompt means I succeeded in resuscitating my patient.

After a few minutes she expressed herself as much relieved. I remained with her some time, and then left careful directions with the husband in case any other unfavorable symptoms should occur. During the next few hours the patieut fainted twice, but she was restored by dashes of water in the face.

This case called to mind what I had recently heard from Doctor Wenger, regarding the use of hypodermic injections. I wrote him, requesting notes of accidents which had fallen under his observation, and he kindly sent me the following reply:

GILMAN, ILL., June 4, 1877.

E. F. INGALS, M.D., Chicago.—*Dear Doctor*—Your favor of the 29th ult. received and contents noted. I am very glad that your attention has been called to the subject of injecting morphine into the system. I have used it myself, but never without some fear; fortunately I have not had any bad results from it, and think I never will. I herewith send you a hasty prepared paper on the subject, which you are at liberty to use as you think best. The last case mentioned was my mother-in-law, the physician a most excellent man; the disappintment, remorse and regret that will follow him through life, is enough to deter any man from ever using the instrument, that knows anything about the case.

Very respectfully yours, E. WENGER.

The paper reads as follows:

That medicine will enter the circulation by absorption from blistered surfaces, and produce its constitutional effects, has been known for a long time, but the introduction of medicine into the system hypodermically, by mechanical means, is, I believe, of rather recent origin. And so popular has this new plan of administering medicine become, that almost every physician is armed with a hypodermic syringe, ready to perform the squirting operation upon the patient for almost every ache or pain, and I believe the profession generally sanction it as a safe and harmless means of relief, especially the young and more enthusiastic.

To condemn the practice as pernicious and unjustifiable, would be rather a bold step just now, but I desire to call the attention of the profession to a few cases that have come under my observation, the result of which have been sufficient to convince me that to inject morphine into the circulation is dangerous, and should never be done when there is any possible means of giving it in any other way.

Case 1. I was called to see a woman in her fifth confinement. On my arrival, found her in convulsions. Another physician had been called, who arrived in a few moments. I at once suggested bleeding. The doctor had just bought a hypodermic syringe (the first I had ever seen,) and suggested the injection of morphine, to which I assented. The effects of the convulsion, or the injected morphine, brought on a heavy stupor, with slow, stertorous breathing, from which she could not be aroused. I delivered her with the forceps. She slept on until she breathed her last.

Case 2. An old gentleman was suffering with rheumatism; he was attended by a promising young physician who had gained some notoriety by the use of the hypodermic syringe. He injected morphine on this occasion, and in a few minutes became alarmed at the condition of his patient. He at once sent for another physician, who, on his arrival, learned the condition of things. Noble-hearted man that he was, to save the young man's reputation, he pronounced it a case of appoplexy. By prompt and persevering treatment, he succeeded in saving the old man's life. He then advised the young man in a friendly way, to be a little cautious how he used the squirt in the future.

Case 3. Mrs. W., aged about 56, had pain in one of her shoulders; in the morning she called the family physician, a man of knowledge and experience. He injected morphine into the arm, and relieved her in a few minutes; in the evening he called again, and found his patient well, but thought to secure a good night's rest, it would be well to inject a little more morphine, which he did, and in thirty minutes his patient was a corpse. The feelings of the doctor can be better imagined than expressed.

I have other cases that I could mention, but these are enough to satisfy me that the hypodermic syringe should never be used where there is any possible way of giving the medicine in any other way. E. WENGER, M.D.

Gilman, Ill., June 1, 1877.

Since writing the above I have inquired of a few of my medical friends and find that several of them have had unpleasant experiences with the use of morphia by hypodermic injections. Two of my friends have had cases almost identical with my own, and doubtless many others have come under the observation of those who have made extensive use of this method of treatment.

I am satisfied that no precaution can be taken which will insure us against accidents from this mode of treatment.

